# Comparing Students’ Engagement in Classroom Education Between China and Germany

**DOI:** 10.3389/fpsyg.2021.754637

**Published:** 2021-10-05

**Authors:** Shu Deng

**Affiliations:** Research Center for Education Development, Beijing Academy of Educational Sciences, Beijing, China

**Keywords:** education, engagement, success, educational achievement, international student assessment

## Abstract

Since the mid-1980s, there has been an academic shift toward students’ involvement in the learning process. A great number of studies have focused on the relationship between student engagement and educational achievement. They have highlighted that appropriate educational input and a supportive classroom environment are necessary, but optimum learning should occur when students are engaged with the curriculum as well as the institution, particularly in higher education institutions. Many scholars claimed that higher levels of engagement will help students deal with academic anxiety and develop a sense of belonging, which may lead to higher academic success. Educational experts and policymakers have begun to propose nationwide and international strategies and programs to promote student engagement in the classroom, which has led to the proposal of well-known programs such as the National Survey of Student Engagement, the UK Engagement Survey, and Program for International Student Assessment. Such engagement-centered international measures have been used across the globe (e.g., Germany) and translated into different languages (e.g., Chinese). Although the findings of relevant studies confirm the effectiveness of engagement on learning achievement, there is still the need to conduct further (cross-sectional) studies considering the implementation of such programs in a different context. The present study is an attempt to review the related literature regarding student engagement among Chinese and German students across a variety of disciplines. The findings suggest that researchers should devote more time and budget to investigate the significance of learner engagement, especially in Germany and China.

## Introduction

Schaufeli et al. (2002) asserted that engagement has been originally implemented in occupational settings, and then, scholars and practitioners have decided to include this concept in the academic environment. They also argued that learning engagement is believed to emphasize personal assets and efficient performance among students. Finn and Zimmer (2012) believed that the concept of student engagement was developed, in the 1980s, as a response to experiences of isolation, boredom, and dropout among students (Schaufeli et al., 2002). Educational courses used to be regarded as successful if students were provided with appropriate materials, and teachers would employ optimum teaching approaches in the classroom. The impact of affective and interpersonal factors as well as classroom environment was claimed to be of great importance in any educational endeavor (Newmann et al., 1992; Pike and Kuh, 2005).

After the proposal of positive psychology, many scholars and practitioners decided to investigate the effectiveness of learners and teachers’ characteristics such as motivation, happiness, enjoyment, and engagement, as the influential factors leading to successful learning outcomes (Gardner and Lambert, 1972; Trowler, 2010; Wang et al., 2021). For instance, Egbert (2020) declared that successful learning happens if only students are eagerly involved in the learning process, and they are passionate about exploiting the learning opportunities in the classroom. Universities and colleges have emphasized the role of student involvement in decision-making activities, given that students’ collaboration with university staff may lead to greater educational achievement (Tchibozo, 2008). In addition, Hunter et al. (2010) proposed that higher education institutions are required to attract and maintain students, provide them with the necessary support, and have them engaged in learning. Consequently, students in higher education environments are regarded as partners, rather than customers, who actively participate in a variety of activities within the university/college (Lowe and El Hakim, 2020).

Ross et al. (2011) claimed that there is a growing tendency in China toward higher education, and therefore, China is regarded as an educational destination for a great number of students in the world. Consequently, they are trying to establish a multitude of private higher education institutions whose major objective is to involve and engage students in the learning process so that the quality of the Chinese educational system can be assessed and recommended reformist ideas can be perceived.

Moreover, the Program for International Student Assessment (PISA) was implemented in different countries so that they can measure students’ performance in reading literacy, mathematics, and science. The German government and especially educational executives were informed of the students’ dropout rate and low educational engagement in the sense of belonging and connectedness as a result of comparing the results of PISA 2000 scores with students from other countries (Ertl, 2006). It is regarded as the beginning of the exploitation of academic and curricular development reform in Germany focusing on providing more appropriate materials and support to students and fostering learners’ autonomy and engagement in the classroom. Consequently, results of PISA 2018 indicated higher levels of engagement and lower levels of skipping or dropout from schools compared to the average scores of students within the organization for economic cooperation and development countries (Education Policy Outlook, 2020).

Given that cross-cultural studies can help practice the existing knowledge in other cultural contexts, findings might contribute to understanding new perspectives regarding the research area and integrating the previous knowledge and the novel information into a more comprehensive concept (Pishghadam et al., 2021). Hence, the present review study aims to investigate learners’ engagement in academic courses and institutions in China and Germany to explore theoretical strengths and weaknesses in this regard. Besides, the assessment of empirical studies within these two contexts should help us provide a clear state about the importance and implementation of the concept of engagement and the probable correlation with other individual, interpersonal, and institutional factors.

## Theoretical Background

According to Trowler (2010), time and effort are the building blocks of student engagement. He proposed that learners and institutions are required to employ necessary resources to promote learning outcomes, enhance learners’ performance, develop institutional fame, and engage learners in the educational process. On the other hand, Teslenko (2019) declared that student engagement is not confined to classroom activities. Higher education institutions have decided to prepare the ground for students to take an active role and voice their ideas in curriculum development, staff recruitment, mental health issues, and even attend strategy development and policy-making meetings. Nonetheless, Lowe and El Hakim (2020) argued that both institutions and students must be wary about the probable disagreements and conflicts, so they should be able to patiently manage such challenges in a mutually respectful environment.

Engagement is defined by a number of scholars and is believed to include various sub-categories. For instance, as one of the prominent figures in this area of research, Reeve (2012) contended that engagement can be defined as a concept encompassing the following modes:

1. Behavioral engagement: This is the major area of interest for scholars and refers to the effort and focus on an educational task leading to taking an active role accordingly;
2. Emotional engagement: It deals with promoting facilitative emotions (e.g., creativity) and diminishing negative emotions (e.g., anxiety);
3. Cognitive engagement: It focuses on meaningful learning through conceptual perceptions in curricular development activities; and
4. Agentic engagement: It refers to deliberate and preemptive involvement in the learning procedure.

Having reviewed the related literature, Egbert (2020) concluded that task engagement can be facilitated through the following enablers:

1. Authenticity: The task is related to their real life;
2. Social interaction: communicating with the teacher or peers to receive proper feedback;
3. Learning support: Required resources should be available and there needs to be enough time and feedback;
4. Student interest: Tasks should be designed according to learners’ interest to engage them;
5. Autonomy: learners’ control over the learning process and teaching approaches; and
6. Task difficulty: The tasks should be designed a little beyond learners’ capabilities so that they perceive the need to make an effort to perform the task successfully.

On the other hand, teachers and instructors believe that developing and maintaining students’ engagement in the classroom is becoming a challenging task these days (Hiver et al., 2021). Furthermore, Egbert (2020) argued that teachers should highlight educational objectives so that learners’ capabilities and demands are taken into account, and then, students are encouraged to be engaged in classroom tasks. Moreover, Xie and Derakhshan (2021) claimed that teachers’ positive communication with learners can result in educational attainment for learners with different characteristics.

**Figure 1:**
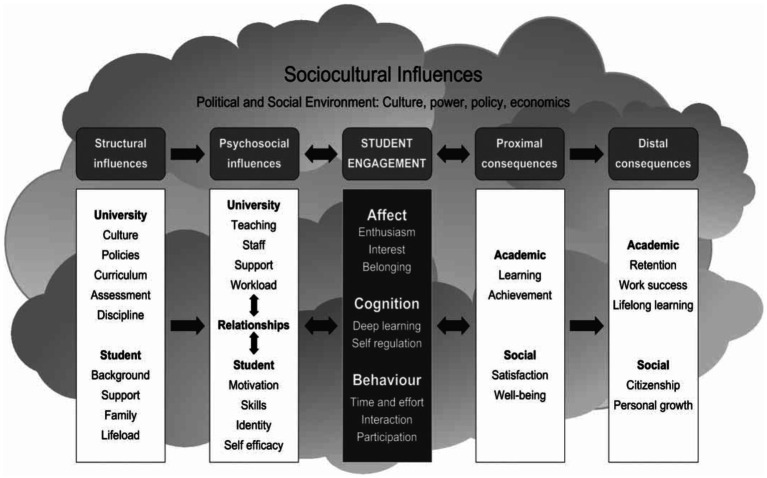
Kahu’s (2013), p. 766 Framework for student engagement in higher education.

Eventually, we should present the conceptual framework proposed by Kahu (2013). It was introduced with an emphasis on the psychological perspective on student engagement, along with the socio-cultural and behavioral views. This framework encompasses institutional and personal factors based on the following sub-categories: socio-cultural, structural, and psycho-social influences as well as proximal and distal consequences (see Figure 1).

## Empirical Studies

### Students Engagement Assessment Instruments

Academic assessment of students’ engagement was proposed by Indiana University, the United States in the early 2000s (Kuh, 2009). They would administer the National Survey of Student Engagement (NSSE), as an institutional instrument to evaluate students’ academic engagement, each year. NSSE was designed so as to measure engagement at curriculum and university levels. It was then followed by some other related measures in different countries, for instance, the UK Engagement Survey and the Australian Survey of Student Engagement(Lowe and El Hakim, 2020). In 2007, a panel of PhD candidates along with a visiting professor from Tsinghua University intended to translate NSSE into Chinese. They further developed the NSSE-C instrument as a standardized measure of students’ engagement in the Chinese context.

In this regard, Ross et al. (2011) argued that such contextualized assessment programs can effectively measure and improve the quality of education across nations, that is, because they help collect data on students’ experiences throughout their education that might lead to expression of their ideas and learning achievement accordingly. For instance, the Chinese version of NSSE assessment survey (NSSE-C) was used to collect data regarding student engagement from students at Tsinghua University in China in 2009. Ross et al. (2011) reported that the findings of this study led to the development of some influential programs such as holding discussions within the university context focusing on the establishment of student-faculty collaborations and promoting undergraduate teachers’ professional knowledge.

On the other hand, Martin et al. (2015) conducted another seminal cross-national survey on student engagement among high students in the United States (*n*=975), Canada (*n*=562), England (*n*=1,558), Australia (*n*=33,778), and China (*n*=3,753). They employed the Motivation and Engagement Scale for High School developed by Martin (2010), which includes 44 items scored based on a 7-point Likert scale. They found that there is a consistency in the measurement of student engagement and motivation among these different regions since the normality distribution and reliability indices were similar. Therefore, Motivation and Engagement Scale is regarded as a generalizable cross-cultural instrument in this regard. Finally, they asserted that motivation and engagement were regarded as distinct concepts by the study population across various cultures.

### Student Engagement Across Various Disciplines

Hiver et al. (2020) developed an assessment instrument to measure cognitive, social, affective, and behavioral dimensions of students’ engagement in language learning classroom. The proposed scale can help evaluate involvement and persistence among students while completing a task. Moreover, Liu and Flick (2019) attempted to investigate the relationship between class engagement and academic performance provided that learners’ psychological needs are satisfied. For this purpose, 573 accounting students from two universities in China were selected to participate in this study. The questionnaire included 5 items on student effort, 7 items on class involvement, and 7 items on task persistence, which were scored using a 7-point Likert scale. They concluded that competence and relatedness were the two psychological needs directly related to classroom engagement. On the other hand, effort and persistence were associated with students’ academic performance.

Meng et al. (2018) examined the effect of information and communication technology (ICT) engagement and students’ academic performance among high schools students in China (*n*=9,841) and Germany (*n*=6,504). The data were obtained from the PISA administered in 2015. They further concluded that there was a significantly positive relationship between learners’ perceived autonomy and academic achievement in science, reading, and mathematics among Chinese and German students. However, there were some inconsistencies among these students of different cultures. For example, Chinese students believed that ICT interest had a positive impact on their achievement, while German students reported a negative impact accordingly. In the end, Meng et al. (2018) argued that the collectivist nature of Chinese cultural compared to the individualist framework of German culture might be regarded as the predictors of such incongruity among students in this study.

## Implications and Suggestions

Hiver et al. (2021) asserted that higher learner engagement scores indicate their responsibility for their own learning and academic success. They claimed that communicative language teaching paradigms welcomed students’ involvement as a crucial factor in academic success and language development. Therefore, it is recommended to investigate students engagement from a variety of perspectives among language learners to identify the effective factors and lead learners toward enduring success in their education.

Moreover, Ross et al. (2011) believed that conducting cross-cultural evaluations of student engagement (e.g., NSSE) can result in the identification of actionable areas of higher education measures, which helps (higher education) institutions improve their curriculum and collaborations with learners. Consequently, it is highly suggested to assess students’ engagement from different cultures using fresh data or even classified data collection measures such as PISA. In this regard, Meng et al. (2018) argued that economic relationships between different countries could prepare the ground for cross-cultural investigation of various disciplines accordingly. For instance, China and Germany are considered as the major economic partners these days and there is a growing number of visiting students between the two countries. Nevertheless, there are very few cross-cultural studies in academic or business areas of research, which might help develop a better perception of the two cultures leading to deeper and more successful mutual relationships in the future.

## Author Contributions

The author confirms being the sole contributor of this work and has approved it for publication.

## Funding

This study was supported by Beijing Office for Education Sciences Planning- “Research on Innovation and Reform of Digital Teaching Mode in German Universities” (Grant No. AADB2020180).

## Conflict of Interest

The author declares that the research was conducted in the absence of any commercial or financial relationships that could be construed as a potential conflict of interest.

## Publisher’s Note

All claims expressed in this article are solely those of the authors and do not necessarily represent those of their affiliated organizations, or those of the publisher, the editors and the reviewers. Any product that may be evaluated in this article, or claim that may be made by its manufacturer, is not guaranteed or endorsed by the publisher.
